# Willingness to perform induced abortion and associated factors among graduating midwifery, medical, nursing, and public health officer students of University of Gondar, Northwest Ethiopia: institution based cross sectional study

**DOI:** 10.1186/s12884-020-03382-0

**Published:** 2020-11-10

**Authors:** Mihretu Molla Enyew

**Affiliations:** Department of Clinical Midwifery, School of Midwifery, College of Medicine and Health Sciences, University of Gondar, PO box 196, Gondar, Ethiopia

**Keywords:** Willingness, Induced abortion, Graduating students, Ethiopian abortion law

## Abstract

**Background:**

In developing countries, abortion is often unsafe and a significant cause of maternal morbidity and mortality accounting for about 8% (4.7–13.2%) of maternal mortality worldwide. Internationally, safe abortion services are recognized as reducing maternal mortality, and liberalized abortion laws are associated with reduced mortality resulting from unsafe abortion procedures. However, health care providers have moral, social and gender-based reservations that affects their willingness towards providing induced abortion services. The purpose of this study was to assess willingness to perform induced abortion and associated factors among graduating Midwifery, Medical, Nursing, and Public health officer students of University of Gondar.

**Methods:**

Institution based cross sectional study was conducted from March 29 to May 30, 2019. All graduating students available during data collection period were considered as study population. Stratified simple random sampling technique was used to select 424 study participants. Pre tested, semi- structured, self-administered questionnaire was used to collect data. Data analysis was done using SPSS version 20. Ethical clearance was obtained from School of midwifery under the delegation of institutional review board of university of Gondar.

**Results:**

Two hundred ninety students out of 424 students were willing to perform induced abortion for indications supported by Ethiopian abortion law, making a proportion of 68.4% (95%Cl: 64.2, 72.9). Sex (Being male (AOR = 4.89, 95%CI: 3.02, 7.89)), religion (being orthodox than protestant (AOR = 10.41, 95%CI: 3.02, 21.57)), being Muslim than protestant (AOR = 5.73, 95%CI: 1.37, 15.92)) and having once or less a week religious attendance (AOR = 2.00, 95% CI: 1.20, 3.34) were factors associated with willingness towards performing induced abortion.

**Conclusions:**

According to this study willingness of students towards providing induced abortion services was good. However female students, protestant followers and those students with more than once a week religious attendance should be encouraged to support women’s access to induced abortion services by referring them to other health care professionals willing to provide induced abortion services.

**Supplementary Information:**

The online version contains supplementary material available at 10.1186/s12884-020-03382-0.

## Background

Over 25 million unsafe abortions occurred every year worldwide, the majority (97%), occurred in developing countries, attributing 4.7–13.2% of maternal deaths annually [1, 2]. In Ethiopia about 36% of adolescents, 39% of women within 20–24 years of age, 54% of women within 25–29 years of age and 78% of women who are 35 and older years of age commit clandestine abortion which is potentially unsafe [3]. Induced abortion (safe termination) services are recognized as key interventions in reducing maternal mortality and morbidity associated with unsafe abortion procedures [4, 5]. However, health care providers in Sub-Saharan Africa (SSA) have moral, social and gender based reservations that affect their willingness towards performing induced abortion [6]. As a result, complication of unsafe abortion is among the leading cause of maternal morbidities and mortalities in the region [7].

The government of Ethiopia revised and liberalized the abortion law in 2005 to increase the accessibility of safe termination services by taking the amount of unsafe abortion and its health detrimental consequences on maternal health [8]. However, a major reduction of morbidity and mortality from the complication of unsafe abortion has not yet been achieved [9]. The shortage of health care providers who can provide comprehensive abortion care (CAC) is still critical and this is again exacerbated by the unwillingness of some health care providers to provide induced abortion services due to various religious, cultural and biological factors [10, 11].

Graduating students’ willingness towards induced abortion provision is an important influence on their intention and capacity to provide induced abortion services during their future careers [12]. Even though there is no concrete evidence in Ethiopia, studies done in Asia and Africa revealed a significant level of unwillingness towards performing induced abortion by graduating students in their future careers. For instance, medical students involved in a study done in Maharastra (India) described fear due to social norms and illegality of the procedure to provide abortion in their future practice [13]. Another study done in South Africa (SA) revealed only 23% of students were willing to perform induced abortions once they are qualified [14]. Thus, this study will have a fundamental role in pinpointing recommendations to develop positive attitude among graduating students towards providing safe abortion care services thereby reducing maternal morbidity and mortality.

## Methods

### Study design and period

Institution-based cross-sectional study was conducted from March 29 to May 30, 2019.

### Study setting

University of Gondar, College of medicine and health sciences located in Gondar city, Northwest Ethiopia. The University of Gondar is one of the oldest and most well-established higher education institutions in the country. There were a total of 805 graduating Midwifery, Medicine, Nursing and public health officer (PHO) students in the College of Medicine and Health Sciences for 2018/19 academic year.

### Characteristics of participants

Proportionally selected graduating midwifery, medical, nursing, and PHO of 2018/19 academic year were included in the study. Students who were not available due to social or medical reasons during the data collection period were excluded.

### Sampling

Sample size was calculated using single population proportion sample size calculation formula. Assuming willingness of students to be 50%, Zα/2 value of 1.96 and marginal error of 5%, Sample size was calculated as follows:

n = (Zα/2)^2^pq/w^2^

n = (1.96)^2^(.5) (.5)/(0.05)^2^

*n* = 385

Adding 10% non-response rate

**N**_**f**_ **= 424**

**Fig. 1:**
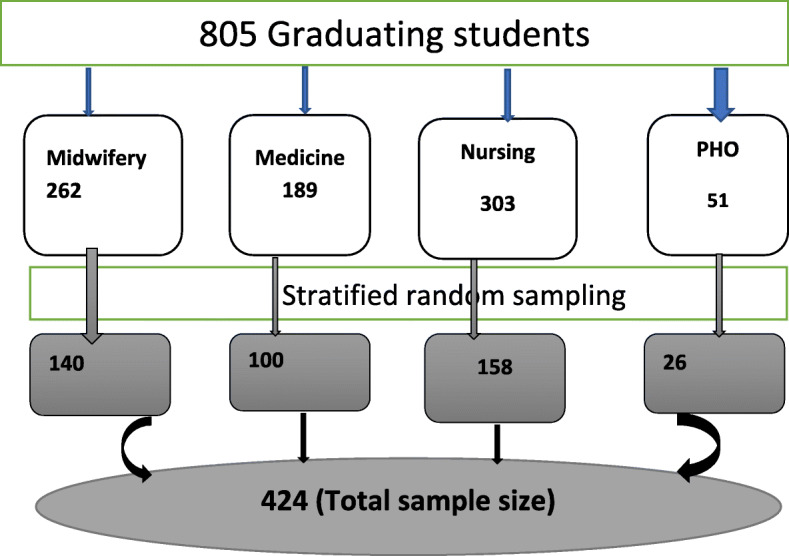
Schematic presentation of enrolment of study participants of willingness to perform induced abortion and associated factors among graduating midwifery, medical, nursing and PHO students of university of Gondar, 2019. N = 424

Stratified random sampling technique was used to select study participants (Fig. 1).

### Variables of the study

Dependent variable

- willingness of graduating students (willing, not willing)

Independent variables

- Age, field of study, marital status, religion, ethnicity, sex, family place of residence, frequency of religious attendance, exposure to sexual practice, exposure to abortion care services

### Operational definitions

#### Willingness of graduating students

Status and level of willingness of students in performing induced abortion following graduation for indications supported by Ethiopian abortion law.

#### Induced abortion

Deliberate termination of pregnancy before viability for reasons of nationally liberalized indications.

#### Liberalized components of induced/safe abortion services

Nationally supported indications to terminate pregnancy such as pregnancy following rape, pregnancy following incest, minority pregnancy, pregnancy endangering the woman’s life and pregnancy complicated by gross fetal defects [15].

### Data collection tools and procedures

Data were collected by self-administered interviews using a semi-structured and pre-tested questionnaire. Six Bsc midwives and two Msc midwives were assigned to collect data and to supervise the data collection process respectively. The questionnaire used to collect data was developed for this study (Additional file 1: Annex).

### Data quality control

The quality of data was assured by proper designing and pre-testing of the questionnaires on 5% of study participants at Debretabor University and by giving training for the data collectors and supervisors before the actual data collection. Every day after data collection, questionnaires were reviewed and checked for consistency and completeness by the supervisors. Data clean up and cross-checking was done before analysis.

### Data processing and analysis

All the questionnaires were checked for completeness, manually and coded, then entered into Epi info 7 and exported to SPSS version 20 software package for further analysis. Descriptive analysis results were presented in the form of table, figure, and text using frequencies and summary statistics such as mean, standard deviation and percentage. Bivariate logistic regression analysis was used to determine the association of each independent variable with the outcome variable and multivariable logistic regression analysis was employed to adjust the influence of various independent variables (confounding effects) on the outcome variable. Odds ratio (OR) with 95% confidence interval (CI) was used to see the association between independent variables and the dependent variable.

## Results

### Socio-demographic characteristics

A total of 424 graduating students were included in the study, making a 100% response rate. The age of the study participants was between 20 and 30 years with mean (±SD) age 23.15 ± 1.77 years (Table 1).

**Table 1:** Socio demographic characteristics of study participants of attitudes and associated factors on induced abortion among graduating midwifery, medical, nursing and health officer students of university of Gondar, northwest Ethiopia, 2018. *n* = 424

Variable	Frequency	Percent (%)
**Age**		
20–24	372	87.7
25–29	42	9.9
30–35	10	2.4
**Sex**		
Male	225	53.1
Female	199	46.9
**Field of study**		
Midwifery	140	33
Medicine	100	23.6
Nursing	158	37.3
Public health officer	26	6.1
**Family place of residence**		
Urban	149	35.1
Rural	275	64.9
**Marital status**		
Single	392	92.5
Married	32	7.5
**Religion**		
Orthodox	339	80
Muslim	64	15
Protestant	21	5
**Ethnicity**		
Amhara	245	57.8
Kemant	47	11.1
Oromo	56	13.2
Tigray	23	5.4
Sidama	47	11.1
Others^a^	6	1.4
**Religious service attendance**		
More than once a week	279	65.8
Once a week or less	145	34.2
**Exposure to sexual practice**		
Yes	124	29.4
No	300	70.6
**Exposure to abortion care services**		
Yes	377	88. 9
No	47	11.1

**Others**^a^**:** *Gurage, Somali, Hadiya, Wolayta, Kembata*

### Willingness of students towards performing induced abortion

**Fig. 2:**
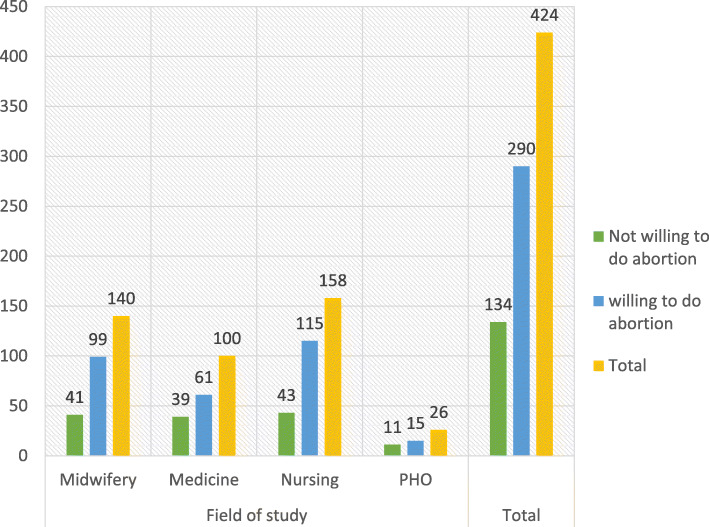
Proportion of willing students to perform induced abortion among graduating midwifery, medical, nursing and PHO students of university of Gondar, 2019. *N* = 4

Of the total 424 study participants, 290 of them were willing to perform induced abortion for indications supported by law, making a proportion of 68.4% (95%Cl: 64.2, 72.9). About 70.7% of midwifery students, 61% of medical students, 72.8% of nursing students and 57.7% of PHO were willing to provide induced abortion services (Fig. 2).

### Factors associated with willingness of graduating students towards performing induced abortion

In bivariate logistic regression analysis, willingness towards performing induced abortion was associated with male sex, single marital status, urban family place of residence, orthodox and Muslim religions and frequent religious attendance.

Among variables found to be significantly associated with willingness towards doing induced abortion using bivariate logistic regression analysis, male sex (AOR = 4.89, 95%CI: 3.02, 7.89), being orthodox than protestant (AOR = 10.41, 95%CI: 3.02, 21.57) being Muslim than protestant (AOR = 5.73, 95%CI: 1.37, 15.92) and less than once a week religious attendance (AOR = 2.00, 95% CI: 1.20, 3.34) were also found to be significantly associated in multivariate logistic regression analysis (Table 2).

**Table 2:** Bivariate and multivariate logistic regression analysis of Factors Associated with willingness towards performing induced abortion among graduating midwifery, medical, nursing and health officer students of university of Gondar, northwest Ethiopia, 2018. n = 424

Variable	Willing to do abortion		COR (95% CI)	AOR (95% CI)	***P*** value
	Yes	No			
**Sex**	Frq	Frq			
Male	189	36	5.09 (3.24, 8.01)	**4.89 (3.02, 7.89)**	**.000**
Female	101	98	Reference		
**Family residence**					
Urban	116	33	2.04 (1.29, 3.23)	1.52(0.91, 2.54)	.108
Rural	174	101	Reference		
**Marital status**					
Single	281	111	6.47 (2.90,14.42)	1.59 (0.58, 4.32)	**.**369
Married	9	23	Reference		
**Religion**					
Orthodox	249	90	16.60(4.78,57.69)	**10.41 (3.02, 21.57)**	**.000**
Muslim	38	26	8.77 (2.34, 32.83)	**5.73 (1.37, 15.92)**	**.017**
Protestant	3	18	Reference		
**Religious attendance**					
≤ once a week	116	29	2.41 (1.50, 3.88)	**2.00 (1.20, 3.34)**	**.008**
> once a week	174	105	Reference		

## Discussion

This study assessed willingness of graduating midwifery, medical, nursing and PHO students towards providing induced abortion services and found 290 students out of 424 students willing to perform induced abortion for indications supported by law, making a proportion of 68.4% (95%Cl: 64.2, 72.9). This finding was in line with findings in California-66.21% [16] and Poland-70% [17].

The result was high when compared with a study done in Argentina-3.34% [18]. This was mainly due to lack of accurate information about legal frameworks of abortion care provision by the Argentinian side. The finding was also high when compared with another study done in Canada [19] where fewer than 30% of students planned to provide any type of abortion. The possible explanation might be Lack of perceived social support for providing CAC and lack of interest to specialize in health care fields containing abortion by Canadian students. The result was also high when compared with a study done Turkey-50.1% [20] and Iran-43.7% [21] respectively. This difference might be due to religious preferences as these two countries are being governed by strict Islamic doctrines. The magnitude was again high when compared with the finding Ireland-58.8% [22]. This might be due to the difference in the year of study of students as the later were not graduating students.

Male students were about five times (AOR = 4.89, 95%CI: 3.02, 7.89) more likely to provide induced abortion services than their female counterparts. This finding is contrary to the findings of previous studies done in Iran [23]. The possible explanation might be long-lasting male dominance in every aspects of participation in our country when compared with Iran.

Concerning students’ religion, being orthodox were about ten times (AOR = 10.41, 95%CI: 3.02, 21.57) more likely to show willingness to induced abortion services than being protestant. Being Muslim was also about six times (AOR = 5.73, 95%CI: 1.37, 15.92) more likely to show willingness to induced abortion services than being protestant. This finding is in line with the findings of the studies done SSA [6], SA [14], Iran [21] and Ethiopia [10]. This might be tailored to the fact that religious restrictions of newly introduced Protestantism might have been practiced better than those of relatively older doctrines.

Those students who had religious attendance less than once a week irrespective of the type of religion were two times (AOR = 2.00, 95% CI: 1.20, 3.34) more likely to show willingness to induced abortion services than those who had more than once a week religious attendance. This finding was consistent with the finding of the study done in Iran and Chile (20, [15]). The possible explanation might be the more students attend religious activities the more they become conservative in providing induced abortion services as they might directly link it with doing sin and vice versa.

## Conclusions

According to this study willingness of graduating students towards performing induced abortion was good when compared with other findings. However female students, protestant followers and those students with more than once a week religious attendance should be encouraged to support women’s access to induced abortion services by referring them to other health care professionals willing to provide induced abortion services.

## Supplementary Information

**Additional file 1.** Annex Questionnaire to assess Willingness to perform induced abortion and associated factors among graduating Midwifery, Medical, Nursing, and Public health officer students of University of Gondar, Northwest Ethiopia.

## Data Availability

All data generated or analysed during this study are included in this published article and in its supplementary information files.

## Abbreviations

AOR: Adjusted Odds Ratio
CAC: Comprehensive Abortion Care
CI: Confidence Interval
COR: Crude Odds Ratio
EMwA: Ethiopian Midwives Association
Frq: Frequency
IRB: Institutional Review Board
Km: Kilo Meters
N_f_: Final Sample size
PHO: Public Health Officer
SA: South Africa
SPSS: Statistical Package for Social Sciences
SSA: Sub- Saharan Africa
ST: Safe Termination
UNICEF: *United Nations International Children’s Emergency Fund*
WHO: *World Health Organization*
