# Supplementary material for: Willingness to perform induced abortion and associated factors among graduating midwifery, medical, nursing, and public health officer students of University of Gondar, Northwest Ethiopia: institution based cross sectional study
Source: BMC Pregnancy Childbirth. 2020 Nov 10;20:676. doi: 10.1186/s12884-020-03382-0 (PMC7654038; doi:10.1186/s12884-020-03382-0)
Supplement: Supplementary file 1 — Additional file 1. Annex Questionnaire to assess Willingness to perform induced abortion and associated factors among graduating Midwifery, Medical, Nursing, and Public health officer students of University of Gondar, Northwest Ethiopia. [file 12884_2020_3382_MOESM1_ESM.docx]

**Annex**

**Questionnaire to assess Willingness to perform induced abortion and associated factors among graduating Midwifery, Medical, Nursing, and Public health officer students of University of Gondar, Northwest Ethiopia**

Hello! My name is --------------. I am professional midwife working with Ato Mihretu Molla, Lecturer of midwifery at university of Gondar. The main aim of this research project is to assess Willingness to perform induced abortion and associated factors among graduating Midwifery, Medical, Nursing, and Public health officer students of University of Gondar, Northwest Ethiopia

I have identified you as a study participant hoping that you would be willing to help provide me with some information.

I have several questions which I would like to ask you, if you have the time and are willing. Participation in this study is completely on voluntary basis and you have the right to refuse from participating either in the beginning or in the middle.

Your responses will be kept confidential and there will be no way of linking your individual responses to the final results of the study findings.

I would like to inform you that the responses that you provide to the questions are very essential, not only, for the successful accomplishment of the study, but also for producing relevant information which will be helpful in improving maternal health.

Would you participate in responding to the questions in this questionnaire?

1. Yes B. No

Questionnaire Code __________
Date of data collection ----------------------------------------
Name of data collector--------------------------------------- signature--------------------
Name of supervisor------------------------------------------- signature--------------------.

**Part I: SOCIODEMOGRAPHIC INFORMATION**

| No. | Questions | Coding categories | Code |
| --- | --- | --- | --- |
| Q101 | Age (years) |  |  |
| Q102 | Sex | Male | 1 |
|  |  | Female | 2 |
| Q103 | Field of study | Midwifery | 1 |
|  |  | Medicine | 2 |
|  |  | Nursing | 3 |
|  |  | PHO | 4 |
| Q104 | Family place of Residence | Urban | 1 |
|  |  | Rural | 2 |
| Q105 | Marital status | Single | 1 |
|  |  | Married | 2 |
|  |  | Divorced | 3 |
|  |  | Widowed | 4 |
| Q106 | Religion | Orthodox | 1 |
|  |  | Muslim | 2 |
|  |  | Protestant | 3 |
|  |  | Catholic | 4 |
|  |  | Others (specify) |  |
| Q107 | Ethnicity | Amhara | 1 |
|  |  | Kemant | 2 |
|  |  | Oromo | 3 |
|  |  | Tigrie | 4 |
|  |  | Others (specify) | 5 |
| Q108 | Frequency of religious service attendance | More than once a week | 1 |
|  |  | Once a week or less | 2 |
| Q109 | Have you been Exposed to sexual practice? | Yes | 1 |
|  |  | No | 2 |
| Q110 | Have you been exposed to abortion care services | Yes | 1 |
|  |  | No | 2 |

**Part II: willingness related Questions**

| No | Questions | Coding category | Codes |
| --- | --- | --- | --- |
| Q201 | Are you willing to provide induced abortion service for indications supported by Ethiopian abortion law? | Willing | 1 |
|  |  | Not willing | 2 |
| Q202 | For question 201 if your answer is code 2 what are your reasons (more than one choice is possible) | Due to religious impact | 1 |
|  |  | Due to cultural impact | 2 |
|  |  | Due to my own belief | 3 |
|  |  | Because it is out of norm | 4 |
|  |  | Others (specify) | 5 |

**If you have any additional opinion about this study you can explain it below.**

___________________________________________________________________________________________________________________________________________________________________________________________________

**Thank you for your time and cooperation!!**
